# Supplementary material for: Carbapenem-Resistant Enterobacteriaceae (CRE) among Children with Cancer: Predictors of Mortality and Treatment Outcome
Source: Antibiotics (Basel). 2023 Feb 17;12(2):405. doi: 10.3390/antibiotics12020405 (PMC9952844; doi:10.3390/antibiotics12020405)
Supplement: Supplementary file 1 [file antibiotics-12-00405-s001.zip › antibiotics-2188576-supplementary.pdf]

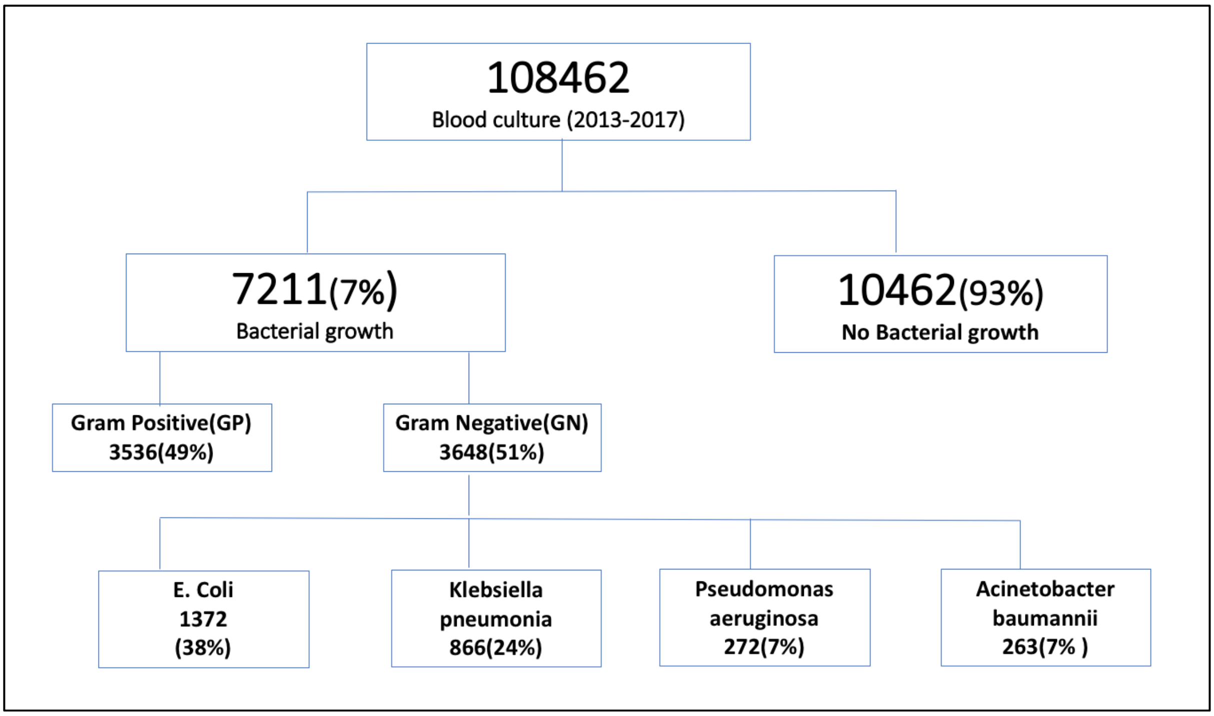

Figure S1: The numbers of blood cultures and the number of positive blood cultures during study period (2013-2017).

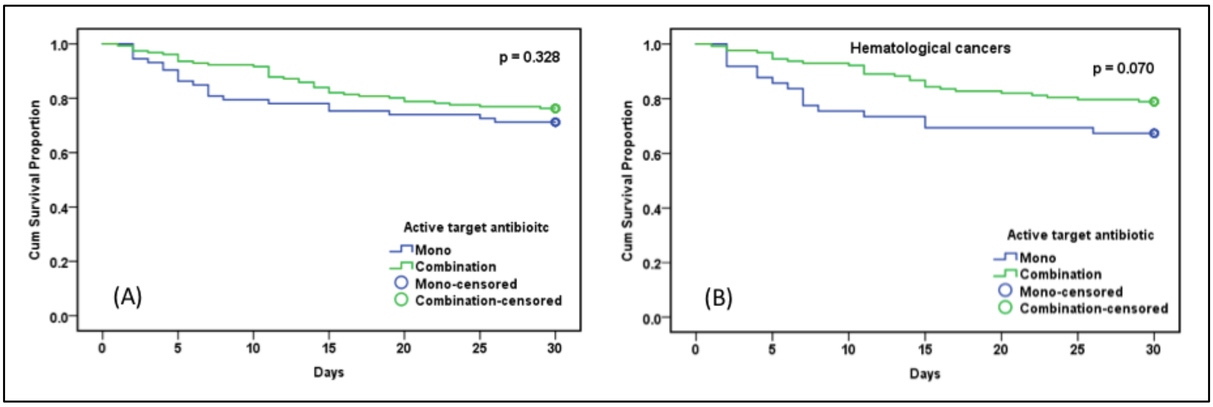

Figure S2: Impact of combination antibacterial treatment versus monotherapy on day 30 mortality (A) among the whole group hematological and solid malignancies (B) patients with hematological malignancies only.
